# Experimental and Computational Studies on the Scattering of an Edge-Guided Wave by a Hidden Crack on a Racecourse Shaped Hole

**DOI:** 10.3390/ma10070732

**Published:** 2017-07-01

**Authors:** Benjamin Steven Vien, Louis Raymond Francis Rose, Wing Kong Chiu

**Affiliations:** 1 Department of Mechanical and Aerospace Engineering, Monash University, Clayton 3800, Australia; wing.kong.chiu@monash.edu; 2 Defence Science & Technology Group, Fishermans Bend 3207, Australia; francis.rose@dsto.defence.gov.au

**Keywords:** Lamb waves, structural health monitoring, edge crack, racecourse shaped hole

## Abstract

Reliable and quantitative non-destructive evaluation for small fatigue cracks, in particular those in hard-to-inspect locations, is a challenging problem. Guided waves are advantageous for structural health monitoring due to their slow geometrical decay of amplitude with propagating distance, which is ideal for rapid wide-area inspection. This paper presents a 3D laser vibrometry experimental and finite element analysis of the interaction between an edge-guided wave and a small through-thickness hidden edge crack on a racecourse shaped hole that occurs, in practice, as a fuel vent hole. A piezoelectric transducer is bonded on the straight edge of the hole to generate the incident wave. The excitation signal consists of a 5.5 cycle Hann-windowed tone burst of centre frequency 220 kHz, which is below the cut-off frequency for the first order Lamb wave modes (SH1). Two-dimensional fast Fourier transformation (2D FFT) is applied to the incident and scattered wave field along radial lines emanating from the crack mouth, so as to identify the wave modes and determine their angular variation and amplitude. It is shown experimentally and computationally that mid-plane symmetric edge waves can travel around the hole’s edge to detect a hidden crack. Furthermore, the scattered wave field due to a small crack length, *a*, (compared to the wavelength *λ* of the incident wave) is shown to be equivalent to a point source consisting of a particular combination of body-force doublets. It is found that the amplitude of the scattered field increases quadratically as a function of *a/λ*, whereas the scattered wave pattern is independent of crack length for small cracks *a << λ*. This study of the forward scattering problem from a known crack size provides a useful guide for the inverse problem of hidden crack detection and sizing.

## 1. Introduction

This paper investigates the scattering of edge-guided waves by a hidden crack as a promising approach for crack detection and sizing in hard-to-inspect locations, motivated by the recent work of Doherty and Chiu [1,2]; see also [3,4,5]. Both experimental and computational investigations were conducted to determine the scattered wave patterns and amplitudes due to the presence of a small crack, when impinged by incident symmetric edge-guided waves. Characterisation of the forward scattering problem is a prerequisite for an inverse scattering approach, as the basis for novel quantitative inspection in structural health monitoring (SHM). 

SHM is a crucial element to regularly monitor structural components for cost effective structural integrity management. Unitised components are a new innovative structural design to improve aircraft performance through weight reduction and fuel efficiency increase [6]. However, reliable detection in this sophisticated design is a significant challenge for SHM. Conventional methods, such as eddy current techniques, are no longer suitable to detect hidden cracks, or cracks in hard-to-inspect locations, because disassembling the components is impossible or too time-consuming [7]. Therefore, there is a need for novel inspection methods to complement the improvements in new complex manufacturing designs.

There is significant interest in using Lamb wave propagation for SHM in such cases due to their large wide-area coverage with low attenuation [8]. However, unlike bulk waves [9,10,11,12,13], it is generally difficult to analytically solve propagating and scattered Lamb wave problems. Hence, experimental and computational studies are required to explore possible approaches for exploiting Lamb wave propagation for crack detection and quantification. Many previous studies [1,2,3,4,5,8,14,15,16,17,18] have investigated the use of low-frequency Lamb wave propagation to detect different type of defects in simple and complex structures. Previous studies [7,19] have investigated creeping waves for hidden crack detection, which involved characteristic length scales where the incident bulk wave is smaller than the crack length (*λ < a*) and hole diameter (*λ << d*). More recently, Doherty and Chiu [1,2] have indicated the possibility that the Lamb wave scattering phenomenon due to the defect can be utilised to characterise the damage for a hard-to-inspect fuel vent hole in the wing spar of an ageing aircraft. However, they did not explore further the potential of scattered wave measurements for quantitative crack characterisation. 

This paper continues and extends recent work [3,4,5,15,20] aimed at using edge-guided waves to detect edge cracks that are located on the blind side of holes (or cut outs) from the viewpoint of conventional inspection techniques. It is highly advantageous to utilise edge-guided waves for SHM, because they do not decay with propagation distance on a straight boundary. However, edge-guided waves propagating around the curved surface will decay at a rate dependent on *d/λ* and Poisson’s ratio. The present work involves edge-guided waves that impinge on a crack at the upper surface of a racecourse shaped hole. Cracking at fuel vent holes of that shape has occurred in practice [21,22], and presents a significant challenge for regular inspection. The scattered wave pattern, and amplitude for various crack lengths, will be reported on and compared to the previous findings for the simpler case of edge cracks along a straight or circular edge [3,4,20].

In the case of bulk waves, it is known that scattering by an infinitesimal crack is equivalent to the wave field from a particular combination of body-force doublets [23]. The Lamb wave scattering by a small edge crack is expected to have similar force doublet equivalents [3,4]. This point source equivalence suggests that the scattering pattern should be relatively independent of crack size, but the amplitude should increase quadratically with increasing *a/λ.* These expectations are indeed confirmed in the present work for a hidden crack on the boundary of a racecourse shaped cut out. This forward scattering study for a known crack length and location is a necessary prerequisite to tackling the practical inverse problem of quantifying and detecting the crack size, based on scattered wave field measurements.

## 2. Background

The fundamental theory for Lamb waves leads to the Rayleigh-Lamb frequency Equations (1,2):

(1) $$\frac{\tan\left(\beta b\right)}{\tan\left(\alpha b\right)}+{\left[\frac{4\alpha \beta \xi^{2}}{\left(\xi^{2}-\beta^{2}\right)}\right]}^{\pm 1}=0\;\{\begin{array}{c} +1\;=symmetric,\;\\ -1=antisymmetric, \end{array}$$

(2) $$\alpha^{2}=\frac{\omega^{2}}{c_{L}^{2}}-\xi^{2}\;and\;\beta^{2}=\frac{\omega^{2}}{c_{T}^{2}}-\xi^{2}$$

where ω and ξ denote the angular frequency and wavenumber of a wave mode and $c_{L}$ and $c_{T}$ are longitudinal and transverse wave speeds, respectively.

Lamb waves are generally dispersive, and if excited at a higher frequency-thickness product, multiple modes will be excited. In the present work, the excitation frequency is selected to be well below the cut-off of 1.53 MHz-mm for the first order symmetrical Lamb wave mode SH1 for aluminium [24]. Hence, the only propagating Lamb waves are the three fundamental modes: the symmetric mode (S0), the shear horizontal mode (SH0), and the antisymmetric mode (A0). For structural health monitoring purposes, the fundamental symmetric wave modes are advantageous due to their simple and uniform through-thickness displacement and stress profile, and essentially nondispersive behaviour [25].

The symmetric modes can be generated by the application of force distributions that are symmetrical with respect to the plate’s mid-plane. Likewise, a symmetric edge-guided wave can also be generated by mid-plane symmetric forces applied along a straight edge. This low-frequency symmetric edge wave can be regarded as the plane stress analogue of the Rayleigh (surface) wave; it is nondispersive, uniformly distributed along the plate thickness, decays with depth, and the corresponding wave speed can be obtained from the Rayleigh wave speed by using the familiar change of elastic constants to convert plane strain results to plane stress [12,26,27]. Edge waves can also propagate around a circular hole (or a curved boundary), but the waves are now (i) dispersive, with the wave speed depending on *d/λ*, and the ratio of hole diameter to wavelength; and (ii) attenuating, because of mode conversion into bulk wave modes [26,27]. 

In order to analyse the scattered wave displacement field, *u_scatter_*, associated with a small hidden crack, a baseline subtraction is employed:

(3) $$u_{scatter}\left(r,\theta ,t\right)\;=\;u_{total}\;\left(r,\theta ,t\right)\;-\;u_{baseline}\left(r,\theta ,t\right)$$

where *u_total_* denotes the response wave field of the cracked structure, and *u_baseline_* denotes the baseline displacement field for the same geometry but without the crack.

The crack length *a* is assumed to be much smaller than the wavelength λ of the incident wave, i.e., *a << λ*. For this crack size limitation, the incident field can be expected to consist primarily of the edge-guided wave. The contribution of propagating bulk wave modes along the edge is negligible. It is anticipated that scattering of the Lamb wave should share similar features to the previous Lamb wave studies on a hole and edge crack problem [3,4,5,15,20]; viz the scattered field can be expected to be like that of a point source located at the crack tip, and with the strength of the point source being proportional to crack length squared. This point source equivalence also suggests that the scattering pattern should be relatively independent of crack size.

## 3. Computational Procedure

**Figure 1:**
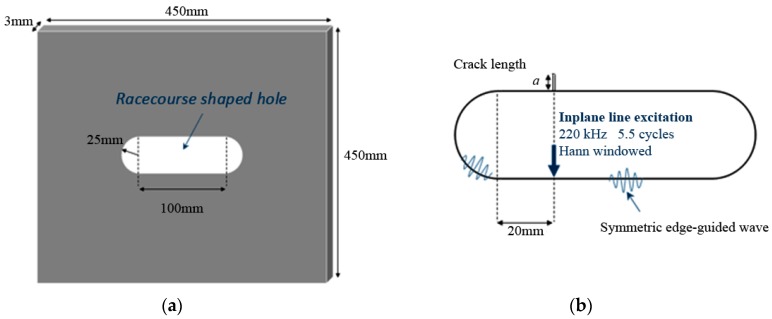
(**a**) Model configuration of the racecourse shaped hole centre in a 450 mm × 450 mm × 3 mm aluminium plate; (**b**) Close-up diagram of the racecourse shaped hole showing excitation and crack location.

In the computational study, ANSYS 15.0 (ANSYS, Inc., Canonsburg, PA, USA) is used as the finite element (FE) computational analysis tool to simulate wave generation and propagation in a 450 mm × 450 mm aluminium plate of 3 mm thickness (density 2700 kg/m^2^, Poisson ratio of 0.33 and Young modulus of 69 GPa). A through-thickness racecourse shaped hole is located at the centre (225 mm, 225 mm) of the plate, as indicated in Figure 1. This hole shape is modelled by two parallel straight lines connected by semi-circles of 25 mm radius at the ends, and the total length of the hole is 150 mm.

The crack is located on the upper straight boundary of the hole, as shown in Figure 1b. The plate is discretised into 0.5 mm 8-node linear hexagonal elements which satisfy the requirement of 10 elements per *λ* for accurate modelling [28]. The time step is set at 0.02 µs, which satisfies the standard stability criterion for explicit time integration of being less than or equal to 0.8 *L/C*, where *L* denotes the smallest element length, and *C* is the fastest wave speed [29]. The defect is modelled as a 0.5 mm width notch to avoid crack face contact. The dependence of scattered amplitude with crack length is investigated by varying the crack size, *a*, from 0.5 mm to 4.5 mm with 0.5 mm increments. The incident edge-guided wave is generated at approximately 8*λ* propagating distances away, with the line force acting in a direction normal to the edge surface. To minimise dispersion, the force excitation signal is chosen to be a 5.5 cycle Hann-windowed tone burst with a centre frequency of 220 kHz. At this centre frequency, the wavelength of S0, SH0 and symmetric edge wave are 22.2 mm, 14.0 mm and 13.4 mm, respectively [24]. It is noted that the wavelength of the circumferential edge wave is slightly larger than the wavelength of symmetric edge waves travelling on a straight boundary; thus, the circumferential edge wave propagates relatively faster. The choice of centre frequency is consistent with an experimental frequency sweep reported below in Section 4. This choice also ensures that the wavelength is sufficiently small to enable the edge waves to propagate around the curved boundary without excessive attenuation. 

Two-dimensional fast Fourier transformation (2D FFT) is performed on the nodes along the line 45° from the crack base edge to create the dispersion curve in order to identify the dominant Lamb wave mode from DISPERSE (Imperial College London, London, UK) [24]. Another 2D FFT scan was performed along the straight boundaries of the hole to determine the symmetric edge-guided wave modal content. The 2D FFT spatial distance is taken at least 3.5 wavelengths away from the crack base to avoid detection of higher non-propagating Lamb waves and approximately 10*λ* distances with 1024 equidistant spatial samples with zero padding [28]. 

**Figure 2:**
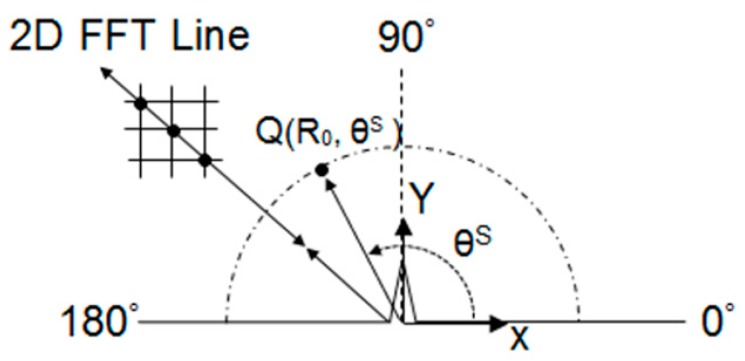
Diagram of a crack on top of the racecourse shaped hole showing 2D fast Fourier transformation (2D FFT) of signal measurement for scattered wave measurements.

The maximum amplitude of the associated signals was used as a measure of the scattered wave amplitude, and to construct the scattering pattern in a polar plot. This is done by taking the maximum peak of the envelope of the analytic signal obtained via a Hilbert transformation, over the time domain signals measured at point Q, which is at a distance approximately 10*λ* away, as shown in Figure 2. 

The scattered wave amplitude is analysed in the region from 0° to 180°, centred at the crack base (refer to Figure 2), and the maximum scattered displacements were measured at 30°, 60°, 90°, 120° and 150° in radial (r) and angular (θ) components. The amplitudes of the back-scatter and forward-scatter edge waves were also analysed by taking the scattering displacements along the hole’s straight boundary.

The scattered S0 and SH0 wave pattern results were then normalised to account for the cylindrical wave decay, which is at a rate inversely proportional to the square root of propagating distance, and relative to the maximum incident edge wave displacement. For the purpose of analysing the wave pattern dependence with crack size, the amplitude is normalised relative to the maximum amplitude of the scattered wave field. This leaky edge wave attenuation over the curved boundary is measured as a function of distance-to-wavelength ratio and briefly reported. This geometry decay is also accounted for in the normalisation process to analyse the scattered edge waves.

## 4. Experimental Procedure

**Figure 3:**
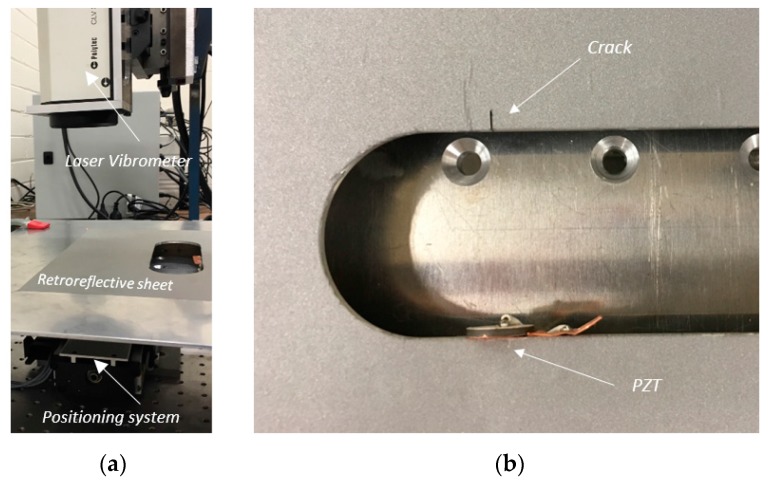
(**a**) Laser vibrometry setup showing a 450 mm × 450 mm × 3 mm aluminium plate with a retroreflective sheet mounted on the positioning system; (**b**) Close-up image of a crack and PZT bonded on the racecourse shaped hole edge.

A 5005H34 aluminium alloy plate with the same geometry as the FE model is employed as the test specimen for the experimental study. The plate is secured on an XY positioning system, as shown in Figure 3, and the experimental rig is mounted on a DAEIL system vibration isolation optical table (DAEIL System Co., Ltd, Cheoin-gu Yongin-si, Korea) to minimise background vibration. The in-plane velocity components of the propagating Lamb waves were acquired by a Polytec CLV 3D automated laser vibrometer (Polytec, Inc., Irvine, CA, USA). In order to enhance the data quality, a Polytec retroreflective sheet is attached to the aluminium plate. A PZ26 piezoelectric transducer; lead zirconate titanate (PZT), of diameter 16 mm and thickness 2 mm was bonded to the straight edge of the hole to generate incident edge-guided waves, as shown in Figure 3. The transducer was nominally placed symmetrically with respect to the plate’s mid-plane, in an attempt to generate only the symmetric modes. However, it was found to be difficult, in practice, to avoid some asymmetry, which results in the generation of weak antisymmetric modes as well. However, because the crack geometry is symmetrical with respect to the plate’s mid-plane, there is no mode coupling due to the scattering process. This means an incident symmetric wave generates only symmetric scattered modes and vice versa for the antisymmetric wave. The symmetric wave modes can be analysed in their dominant in-plane components. However, since the scattered S0 signal is particularly weak, only the SH0 scattered wave amplitude and patterns are analysed in the experimental study. It should be noted that a weak signal from non-dominant modes can still be detected due to the nature of Lamb waves. A frequency sweep between 160 and 250 kHz, at 10 kHz increments, was performed to verify the tuning curve of the PZT [30]. A 2D FFT was performed to show that the symmetric modes are dominant when the PZT is excited at 220 kHz.

In the experimental investigation, the crack is defined as 0.4 mm width notches, which are artificially created to prevent wave transmission through the crack surface as shown in Figure 3. Furthermore, since the width is significantly smaller than the notch length, the effect due to the notch width will be negligible and thus the scattered wave will be predominantly due to the notch length [17,18,31]. The scattered wave patterns of notch length 2.59 mm, 3.49 mm, and 4.64 mm were investigated. The post-processing of data is the same in both computational and experimental investigations.

## 5. Results

### 5.1. Edge-Guided Wave Propagation

**Figure 4:**
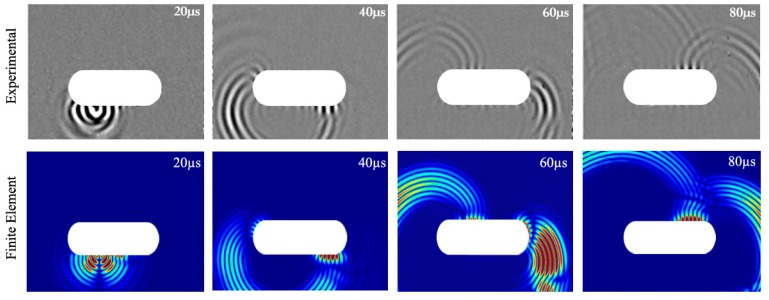
Time snapshot of the experimental wave field in the vertical component and computational total wave field of the edge-guided wave propagating around the racecourse shaped hole at 20, 40, 60 and 80 µs.

The first investigation has shown that a symmetric edge wave can propagate around the curved segment of a racecourse shaped hole for both the experimental and FE study, as shown in Figure 4. This suggests that an excitation of a symmetric edge-guided wave at any point on the hole’s boundary could be used to interact with and detect cracks on the blind side. Furthermore, it is essential to determine the attenuation of these edge-guided waves with propagation distance to enable a quantitative assessment of crack size in hard-to-inspect areas.

**Figure 5:**
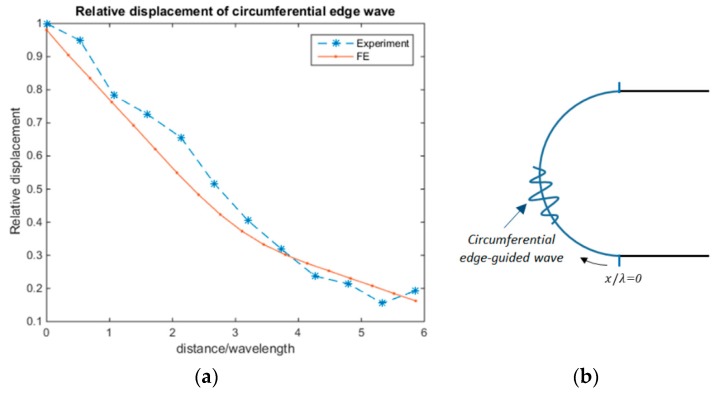
(**a**) The experimental and finite element (FE) attenuation of incident circumferential edge wave displacement over the curved surface; and (**b**) Circumferential edge wave measurements taken on the curved edge (blue semi-circle) starting at the 6 o’clock position and ending at the 12 o’clock position.

The edge wave amplitude does not decay as it propagates on the straight boundaries. However, for *d/λ* ≈ 3.7, as the edge wave travels along the curve boundaries, it leaks energy into the medium at a rate inversely proportional to a power of 0.55 over propagating distance, as indicated in Figure 5. In the next set of investigations, the FE and experimental edge-guided wave results were normalised to account for this decay.

### 5.2. Scattered Wave Field Due to the Presence of a Hidden Crack

**Figure 6:**
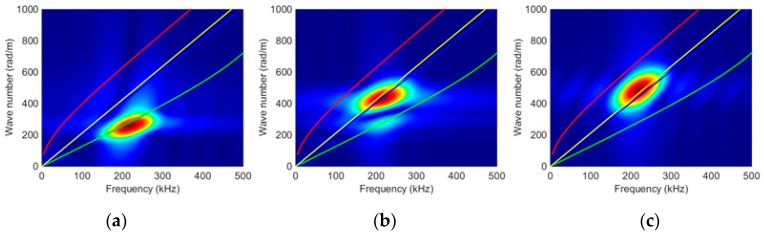
(**a**) Plots of the 2D FFT FE dispersion curve indicating (**a**) a dominant S0 wave in the r component; (**b**) a dominant SH0 wave in the *θ* component; (**c**) a symmetric edge-guided wave along the straight boundary.

The second investigation analysed the presence of a small edge crack and its scattering wave fields. Two-dimensional FFT processing was used to identify the dominant wave modes in conjunction with Lamb-wave dispersion curves from DISPERSE, as shown in Figure 6. The scans taken along the 45° line from the crack base have indicated a dominant scattering S0 and SH0 wave in the radial and angular components, respectively. Thus, the scattered S0 and SH0 wave amplitudes and patterns are analysed in their respective dominant cylindrical components. The 2D FFT scan along the straight boundary of the slot indicated a propagating symmetric edge-guided wave whose speed is similar to the Rayleigh wave speed. 

**Figure 7:**
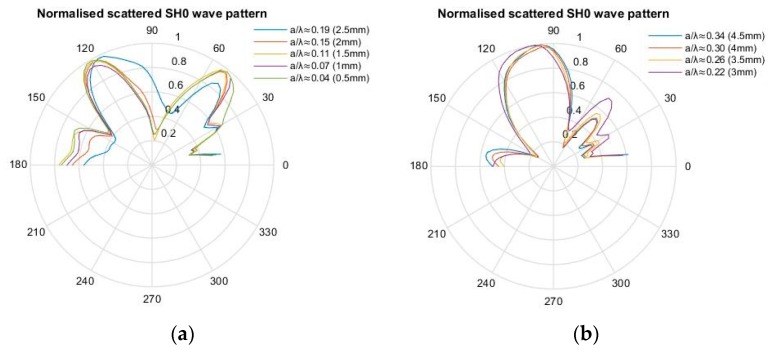
FE-normalised scattered wave polar plot of SH0 waves with respect to the incident wave maximum displacement. (**a**) 0.04 ≤ *a/λ* ≤ 0.19; (**b**) 0.22 ≤ *a/λ* ≤ 0.34.

**Figure 8:**
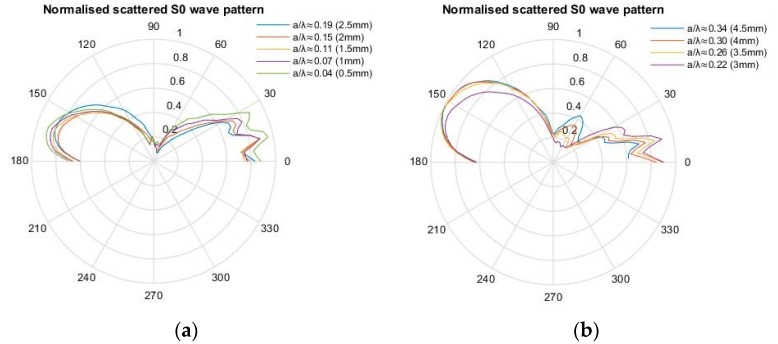
FE-normalised scattered wave polar plot of S0 waves with respect to the incident wave maximum displacement. (**a**) 0.04 ≤ *a/λ* ≤ 0.19; (**b**) 0.22 ≤ *a/λ* ≤ 0.34.

Figure 7 and Figure 8 illustrate the scattering pattern for the SH0 and S0 mode, respectively, for various crack lengths. For *a/λ* ≤ 0.19, the SH0 wave pattern remains symmetrical along the 90° line. However, as crack length increases beyond this limit, the wave pattern is asymmetrical and has a relatively larger back-scatter lobe, which is approximately twice as large as the forward-scatter lobe. There is also an additional contribution of the leaky edge-guided wave in the scattered SH0 wave pattern as shown in Figure 7 in the 150–180° region. 

Figure 8 shows the scattering pattern for S0 waves for various crack lengths. Similar to the SH0 waves, the S0 wave pattern remains independent of the crack length for *a/λ* ≤ 0.19. As the crack length increases, the back-scatter S0 lobe becomes relatively larger than the forward-scatter lobe, and hence, no longer retains its symmetrical pattern. The S0 and SH0 scattered wave pattern results are very similar to those obtained previously for a straight edge [3,4,15].

**Figure 9:**
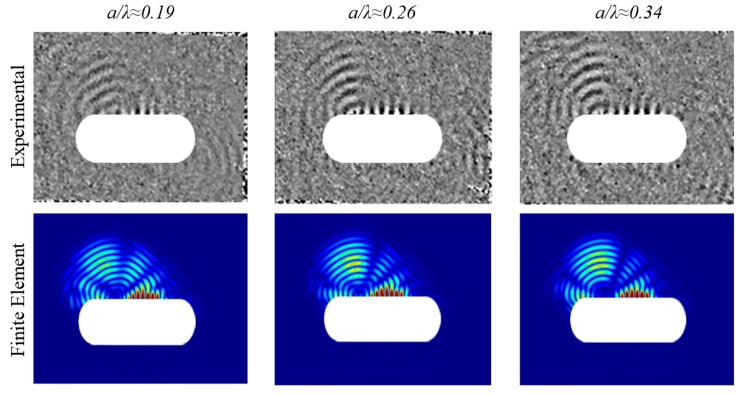
Snapshot of experimental and computational scattered wave field in the angular cylindrical component showing a dominant scattered SH0 wave field at time 70 μs for *a/λ* ≈ 0.19, *a/λ* ≈ 0.26 and *a/λ* ≈ 0.34.

**Figure 10:**
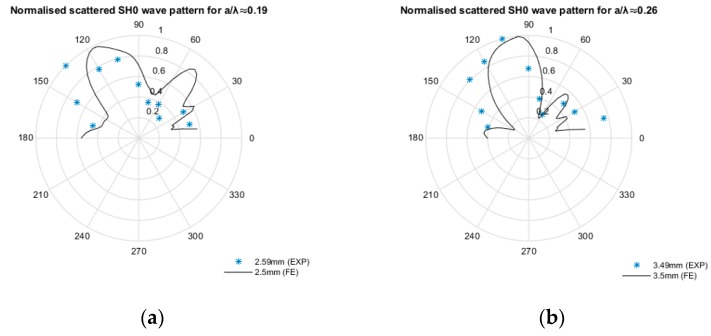

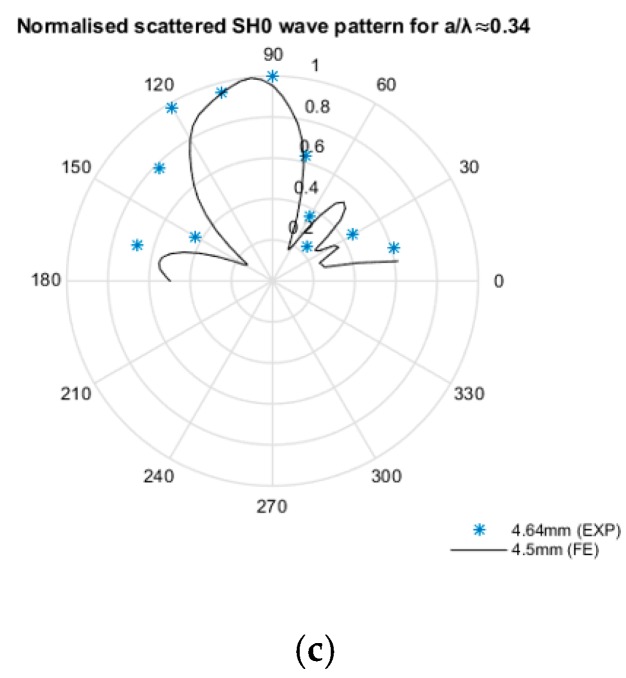
Experimental- and computational-normalised scattered SH0 wave patterns for (**a**) *a/λ* ≈ 0.19, (**b**) *a/λ* ≈ 0.26 and (**c**) *a/λ* ≈ 0.34.

The experimental scattered S0 wave fields are relatively weak and could not be quantified reliably for analysis in this study, therefore only the experimental scattered SH0 waves were compared to the computational results. The *a/λ* ≈ 0.19, 0.26, and 0.34 experimental scattered SH0 wave pattern results also indicated a strong bias back-scatter lobe, and have shown good correlation with the computational results; refer to Figure 9 and Figure 10. 

**Figure 11:**
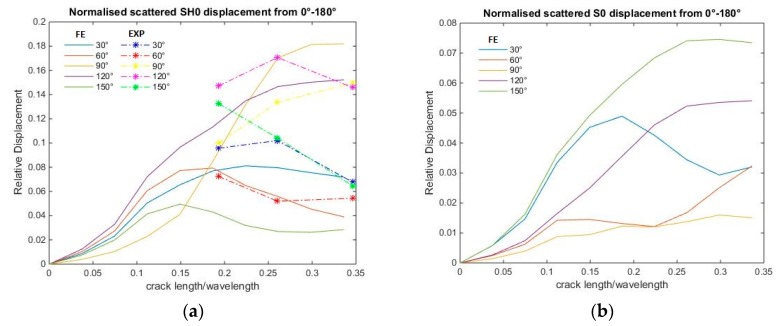
(**a**) FE and experimental results: normalised scattered SH0 wave displacement for various *a/λ*; and (**b**) FE results: normalised scattered S0 wave displacement for various *a/λ* with respect to the incident wave maximum displacement.

**Figure 12:**
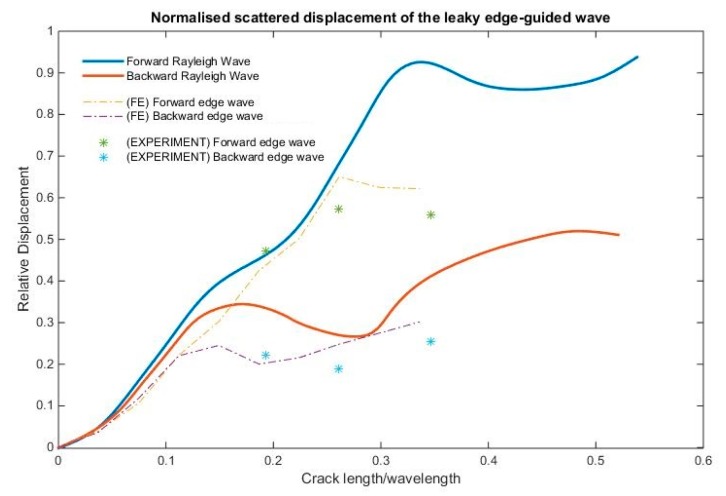
Experimental and computational comparison of the normalised scattered wave displacement of the edge-guided waves with respect to the incident wave maximum displacement, compared with the analytical Rayleigh wave case [13].

The S0 and SH0 scattered wave displacements have indicated an increasing quadratic trend with respect to crack length until approximately *a/λ* ≈ 0.19, as shown in Figure 11. As crack length increases beyond *a/λ* > 0.19, the scattered wave displacement no longer increases with crack length. The forward and backward scattered edge wave maximum displacements are of similar magnitude, and increase as a function of *a^2^* until *a/λ* ≈ 0.11 (Figure 12). The forward scattered edge wave becomes dominant as crack length increases. The experimental scattered wave displacement trends are similar to the computational scattered wave displacement.

The scattered symmetric edge-guided wave amplitude has a similar trend to the analytical result of Mendelsohn et al. [13] for the corresponding plane strain problem as shown in Figure 12. It can be seen that there is reasonably good agreement. The inaccuracy for larger crack sizes is because the FE and experimental results of the incident wave include some contribution from the bulk wave modes, whereas the incident wave for the analytical results is purely Rayleigh wave. Thus, this difference also applies for the scattered wave. 

## 6. Discussion

**Figure 13:**
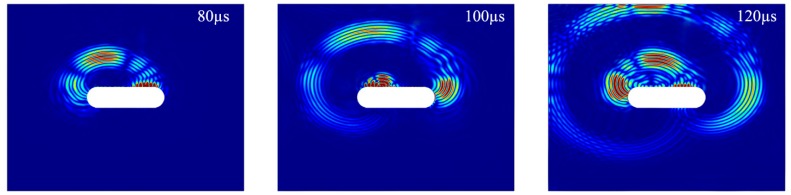
Snapshot of the FE scattered wave field in the angular component due to the presence of a hidden crack.

The FE and experimental results have shown that an incident symmetric edge-guided wave can propagate around a racecourse shaped hole to impinge upon, and thereby detect, a hidden crack. In the present study, the first scattered wave is caused by the incident symmetric edge waves travelling clockwise around the racecourse shaped hole, which constitutes the shortest propagation path to the crack. It is observed that the scattered edge-guided waves propagate around the slot, as shown in Figure 13. Subsequently, a second scattered wave is caused by the incident edge wave travelling counter-clockwise.

It is observed that the scattered edge-guided wave coalescing with the scattered SH0 wave can redirect back to the location of the PZT. It is also possible to generate symmetric edge-guided waves on the boundary by mode conversion of incident symmetric Lamb waves. This suggests that whenever edge-guided waves can be generated around the hole boundary, a hidden crack can be detected. 

Based on the principle of superposition [11], the scattered wave pattern can be produced by applying opposite baseline stress profile as a function of depth on the crack face. However, below a certain *a/λ*, a crack can be approximated as an equivalent point source. The FE results indicated that for a relatively small crack *a/λ* ≈ 0.19, the scattered S0 and SH0 wave patterns are independent of crack length, as shown in Figure 7 and Figure 8, and the scattered wave displacement increases with crack length squared, as shown in Figure 11 and Figure 12. This suggests that the scattered wave pattern due to a crack with length *a/λ* < 0.19 is equivalent to the radiation field produced by a point source. 

**Figure 14:**
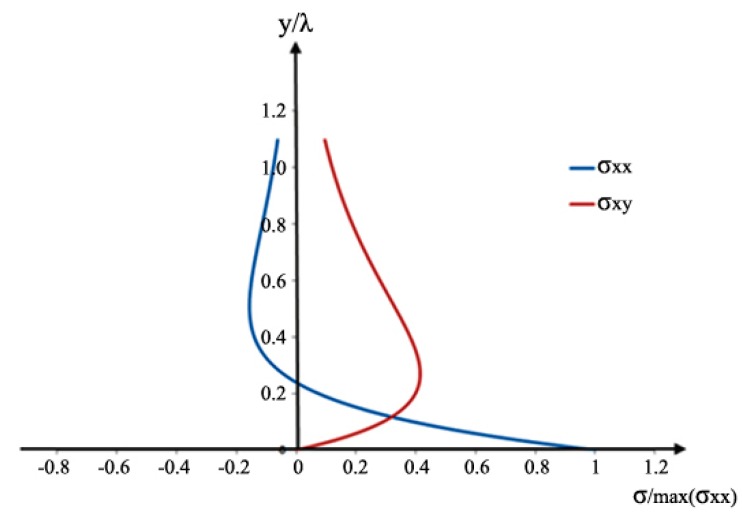
Stress variations associated with the incident edge wave, as a function of depth *y* from the edge.

At approximately *y/λ* = 0.2, the Rayleigh wave exhibits a retrograding particle motion and stress reversal [9,12]. A similar behaviour occurs for the symmetric edge-guided wave particle displacement and stress. For *y/λ* < 0.2, the incident edge wave has a dominant normal stress component σ_xx_, and minimal shear stress component σ_xy_, as shown in Figure 14.

**Figure 15:**
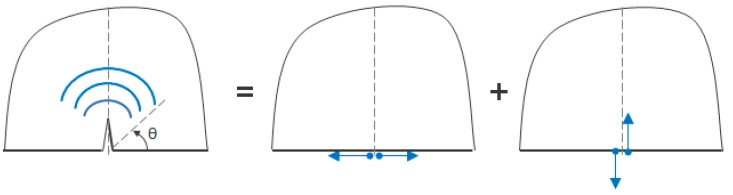
Scattered wave field representation due to the contribution of force doublets associated with the Mode I and Mode II crack opening displacements.

The normal stress gives rise to a Mode I crack opening, whereas the shear stress gives rise to a Mode II crack opening. For small cracks, relative to the incident wavelength, the Mode I crack opening generates a scattered field that is the same as that of a force doublet as shown in Figure 15. This field is symmetrical with respect to θ = 90°. The Mode II crack opening also generates a scattered field that is the same as that for a force doublet, but this doublet, and hence the associated field, is now asymmetric with respect to θ = 90°, as also indicated in Figure 15. 

For *a/λ* < 0.19, the normal stress is much larger than the shear stress, and accordingly, the Mode I contribution dominates the scattered wave field. This explains why the scattering pattern is symmetrical for small cracks, but becomes asymmetrical as crack length increases beyond this value, due to the dominant contribution of shear stress indicated in Figure 7 and Figure 8. Therefore, for *a/λ* < 0.19, the point source representation for a small crack impinged with an incident symmetric edge-guided wave consists primarily of the force doublet representing a Mode I crack opening. 

The results showed that the scattering amplitude appears to increase quadratically, which is in agreement with the theoretical expectation from a quasistatic approximation for long wavelength (low frequency) scattering [32]. In a previous edge crack problem [3], a linear trend was observed for 0.07 < *a/λ* < 0.22. The normal and shear stress variations within 0.07 < *a/λ* < 0.22 (refer to Figure 14), are the reason for the linear relationship of scattering amplitude with crack size. In this hidden crack study, smaller crack lengths were also investigated. Hence, a more obvious quadratic trend can now be observed for the values. 

Beyond the limit *a/λ* = 0.19, the point source representation is no longer valid as the stress profiles and crack opening displacements vary rapidly (Figure 14). It can be seen that the normal stress is a maximum at the surface, decays to zero for *y/λ* ≈ 0.2, and it is negative after that. On the other hand, the shear stress increases to a maximum at *y/λ* ≈ 0.2. This maximum value is less than half the maximum value of the normal stress. These characteristics of the stress distribution serve to explain the features of the asymmetrical wave pattern results.

## 7. Conclusions

This study has shown a method to propagate symmetric edge-guided waves in the shadow zone that can interact with, and hence potentially detect, a small crack at a hard-to-inspect location. In particular, the FE scattered wave direction and scattered amplitude variation with crack size were investigated and shown to be in good agreement with experimental measurements. 

The scattered wave amplitudes and patterns vary with crack size and, thus, serve as a key measurement tool for crack detection and quantification. For a small crack, in the range of *a/λ* < 0.19, the scattering wave pattern is independent of crack size, and the scattering amplitude increases quadratically with the crack size. Furthermore, for such small cracks, the scattering pattern corresponds to that of a force doublet simulating a Mode I crack opening. These results, for the limiting case of a small hidden crack on a racecourse shaped hole, indicate that scattering measurements could be used when tackling the inverse problem of detecting and quantifying hidden cracks, based on the scattered wave field measurements from limited view angles. A more extensive derivation and discussion of the point source equivalence of scattered wave fields due to the presence of a small crack is currently being prepared for publication. It is also pertinent to note that, in practice, changes in operational and environmental conditions can lead to small variations in wave speed [33], and hence in wavelength, which must be taken into account for the inverse problem of estimating crack size.
